# Plasma homocysteine levels and associated factors in community-dwelling adolescents: the EVA-TYROL study

**DOI:** 10.3389/fcvm.2023.1140990

**Published:** 2023-06-23

**Authors:** Nina Gande, Christoph Hochmayr, Anna Staudt, Benoît Bernar, Katharina Stock, Sophia Zollner-Kiechl, Ralf Geiger, Andrea Griesmacher, Sabine Scholl-Bürgi, Michael Knoflach, Raimund Pechlaner, Ursula Kiechl-Kohlendorfer, the (Early Vascular Ageing) EVA Study Group

**Affiliations:** ^1^ Department of Pediatrics II (Neonatology), Medical University of Innsbruck, Innsbruck, Austria; ^2^ Department of Pediatrics I, Medical University of Innsbruck, Innsbruck, Austria; ^3^ Department of Pediatrics III (Cardiology), Medical University of Innsbruck, Innsbruck, Austria; ^4^ Department of Neurology, Medical University of Innsbruck, Innsbruck, Austria; ^5^ Department of Pediatrics, Bruneck Hospital, Bruneck, Italy; ^6^ Central Institute of Clinical Chemistry and Laboratory Medicine Medical University of Innsbruck, Innsbruck, Austria

**Keywords:** cardiovascular risk factors, homocysteine, epidemiology, adolescents, health prevention

## Abstract

**Background:**

Homocysteine (Hcy) has been associated with an adverse cardiovascular risk profile in adolescents. Assessment of the association between plasma Hcy levels and clinical/laboratory factors might improve our understanding of the pathogenesis of cardiovascular disease.

**Methods:**

Hcy was measured in 1,900 14- to 19-year-old participants of prospective population-based EVA-TYROL Study (44.3% males, mean age 16.4 years) between 2015 and 2018. Factors associated with Hcy were assessed by physical examination, standardized interviews, and fasting blood analysis.

**Results:**

Mean plasma Hcy was 11.3 ± 4.5 µmol/L. Distribution of Hcy was characterized by extreme right skew. Males exhibited higher Hcy and sex differences increased with increasing age. Univariate associations with Hcy emerged for age, sex, body mass index, high-density lipoprotein cholesterol, and for factors pertaining to blood pressure, glucose metabolism, renal function, and diet quality, whereas the most important multivariate predictors of Hcy were sex and creatinine.

**Discussion:**

Clinical and laboratory factors associated with Hcy in adolescents were manifold, with sex and high creatinine identified as strongest independent determinants. These results may aid when interpreting future studies investigating the vascular risk of homocysteine.

## 1. Introduction

Adolescence constitutes a critical developmental period during which adverse cardiovascular risk profiles are commonly acquired and consequently a window of opportunity for primary prevention of cardiovascular disease (CVD) (1). Adolescents are already vulnerable to and develop subclinical atherosclerosis upon exposure to traditional cardiovascular risk factors, including arterial hypertension, dyslipidemia, diabetes, and smoking (2). This relationship extends to the sulfur-containing amino acid homocysteine (Hcy), which possesses biological functions in methionine metabolism and associates with subclinical atherosclerosis in children with homocysteinuria (3) as well as community-dwelling, generally healthy youngsters (4–7).

Established causes of hyperhomocysteinemia (HHcy) in children and adolescents include genetic mutations interfering with methionine metabolism, deficiencies of folic acid, vitamin B-6, or vitamin B-12, and end-stage renal disease. Although the causal relation of Hcy to cardiovascular diseases remains uncertain (8, 9), HHcy has further been reported to be embedded within an adverse CVD risk profile in adolescents (10–13). The study objective was to investigate clinical and laboratory factors associated with plasma Hcy levels in a large cohort of community-dwelling adolescents as it may aid in understanding the pathogenesis of CVD and in assessing the vascular risk of homocysteine at this age.

## 2. Materials and methods

### 2.1. Study participants and design

The Early Vascular Aging (EVA) Tyrol Study is a prospective cohort study investigating CVD risk profiles in adolescents, conducted between May 2015 and July 2018 in the federal state of Tyrol, Austria, and the city of Bruneck, South Tyrol, Italy. Generally, all Tyrolean high and vocational schools were informed about the EVA-Tyrol study by the local education authority. Schools and local training companies were then able to apply or were additionally actively contacted and invited to participate in the study by the study team. The aim was to include whole school classes/all apprentices at a company rather than individual volunteers to guarantee an unbiased sample. Inclusion criteria were pupils in 10th grade (mean age 15–16 years) at baseline and 12th grade at follow-up/control group (mean ages 17–18 years). Schools and training companies were randomly assigned to either the intervention or the control group. Exclusion criteria were adolescents attending the military service and persons with impaired power of judgement. Prior disease was no reason for exclusion. Although the study administered a health intervention to a subgroup of participants, none of the participants included in the current analysis received any intervention; hence, the current analysis was observational. The study protocol has been published elsewhere (14). The EVA-Tyrol study was registered at ClinicalTrials.gov (ClinicalTrials.gov Identifier: NCT03929692), was approved by the local ethics committee of the Medical University of Innsbruck (approval number AN 2015–0005 345/4.13), and all the participants and their legal representatives gave informed consent.

### 2.2. Laboratory analysis

Blood samples were drawn after an overnight fast, immediately cooled, and laboratory parameters measured within 24 h after venipuncture. Heparin plasma tubes were used for laboratory measurement of total plasma Hcy and were immediately delivered to the testing facility (iso-certified central institute for medical and chemical laboratory-diagnostics – ZIMCL, Tirol Kliniken, Innsbruck, Austria). Homocysteine was analyzed by The ARCHITECT Homocysteine assay (Abbott Laboratories, Abbott Park, IL), which is a one-step immunoassay for the quantitative determination of total L-homocysteine in human plasma using chemiluminescence microparticle immunoassay technology. Dithiothreitol reduces bound or dimerised homocysteine (oxidized form) to free homocysteine. Free homocysteine is further converted to S-adenosyl homocysteine. The S-adenosyl homocysteine then contests with acridinium-labeled S-adenosyl cysteine for particle-bound monoclonal antibody. Chemiluminescence is resulting from a reaction mixture and measured as relative light units which is an indirect parameter for the amount of homocysteine in the sample (15). Total cholesterol, low-density cholesterol (LDL-C), high-density cholesterol (HDL-C), and creatinine were assessed by standard enzymatic colorimetric assays (Cobas 8,000, Roche Diagnostics, Rotkreuz, Switzerland). Glomerular filtration rate (GFR) was calculated using the Schwartz equation [GFR in ml/min per 1.73 m^2^ = (height in cm/creatinine in mg/dl) × 0.413] (16). Serum glucose and insulin were analyzed by a hexokinase method (Cobas 8,000, Roche Diagnostics, Rotkreuz, Switzerland). Measurements of glycated hemoglobin (HbA1c) were performed by high-pressure liquid chromatography (HA 8,180 T, Menarini Diagnostics, Florence, Italy). The Homeostatic Model Assessment for Insulin Resistance (HOMA) index was calculated as HOMA index = (fasting insulin [mIU/L] × fasting glucose [mmol/L]/22.5). Detailed descriptions of laboratory analysis methods have been published (14).

### 2.3. Genetic testing

Participants with very high homocysteine levels (>97th percentile of the empirical distribution corresponding to >20 µmol/L) were invited for further in-house check-up and genetic testing for the MTHFR polymorphism. Genomic DNA was extracted from EDTA blood samples. The MTHFR-polymorphism (NM_005957.4) c.665C > T (p.Ala222Val; rs1801133) was detected by PCR amplification and Sanger sequencing from genomic DNA using standard PCR protocol (GoTaq G2 DNA Polymerase, Promega) and sequence specific primer for exon 5 (fwd: 5′-TCCCTGTGGTCTCTTCATCC-3′; rev: 5′-CTGGGAAGAACTCAGCGAAC-3′). Sequence analysis was performed using the SeqPilot® software (JSI medical systems GmbH, Ettenheim, Germany).

### 2.4. Anthropometry

Weight and height were measured using medical precision scales and a Harpenden stadiometer (Holtain, Crymych, United Kingdom). Body mass index (BMI) was calculated as the ratio of weight in kg divided by the square of height in meters. Systolic and diastolic blood pressures were measured on both arms after a 5-minute seated rest (automated oscillometric device OMRON M4-I, Omron Healthcare Co., Lake Forest, IL) and the mean of three measurements was used. BMI and blood pressure were assessed using sex- and age-specific grow reference data (17, 18). We opted to use BMI as well as systolic and diastolic blood pressures instead of their z-scores because in our sample high correlations between raw variables and their age- and sex-dependent z-scores were observed.

### 2.5. Assessment of lifestyle risk factors

Lifestyle risk factors were assessed by in-person physician interviews and by lifestyle questionnaires. The adolescents were asked how many minutes of moderate or vigorous activity they do per day according to the American Heart Association definitions of ideal cardiovascular health. Smoking was also categorized according to the American Heart Association recommendations. Current smoking status was defined as non-smoker when never tried smoking or never smoked a whole cigarette and as smoker when tried prior 30 days or smoked regularly. Healthy diet was assessed using the Dietary Approach to Stop Hypertension (DASH) score (19). Moreover, dietary habits were evaluated by the Adolescent Food Habits Checklist (AFHC), which focuses on behavioral aspects of diet in adolescents (20).

### 2.6. Statistical analysis

Baseline characteristics of the study population are given as count (percentage) or mean ± standard deviation. The distribution of Hcy was non-normal by Shapiro–Wilk test and upon histogram inspection, with the latter indicating marked right-skew. Generalized additive models for location, scale, and shape (GAMLSS) were used to fit the age- and sex-conditional distribution of Hcy using the Box-Cox t distribution and fitting penalized varying coefficient terms for age individually by sex (21). Spearman correlation was used to investigate univariable associations of Hcy with demographic, anthropometric, lifestyle, hemodynamic, and complete blood count parameters. Multivariable independent associations of variables significantly univariably associated with Hcy were assessed using L1-regularized linear regression (the LASSO), using the regularization hyperparameter lambda that minimized 10-fold cross-validated mean squared error (22). For this analysis, Hcy was transformed towards normality by an inverse normal transform. As LASSO is focused on predictive performance, we complemented LASSO results with results from a conventional linear regression model including the predictors selected by LASSO to obtain confidence intervals and *p*-values. Analysis was conducted using R 4.2.1 (R Foundation for Statistical Computing, Vienna, Austria) and SPSS 27 (IBM, Chicago, USA). *p*-values are two-sided and an alpha level of 0.05 is used.

## 3. Results

The EVA-Tyrol study included 2,102 participants in its observational arm, 2,046 of which were at most 19 years old, and 1,900 had plasma Hcy measurements available and formed the study population. Participants were on average 16.4 ± 1.10 (mean ± standard deviation) years old and 44.3% were male. Characteristics of the study population are shown in Table 1.

**Table 1:** Characteristics of the study population.

Characteristics	Total *n* = 1,900
Demographics and anthropometrics	
Male sex	841 (44.3)
Age, years	16.42 ± 1.10
BMI, kg/m^2^	21.89 ± 3.56
Lifestyle factors	
Physical activity, minutes per day	53.14 ± 41.45
Smokers, %	578 (29.8)
Hemodynamics	
BP systolic, mmHg	122.77 ± 11.67
BP diastolic, mmHg	71.16 ± 7.59
Lipid parameters	
Total plasma homocysteine, µmol/L	11.33 ± 4,48
Total cholesterol, mg/dl	159.05 ± 30.12
HDL-C, mg/dl	58.40 ± 13.46
LDL-C, mg/dl	93.56 ± 26.18
Glucose metabolism	
Glucose, mg/dl	77.05 ± 9.15
Insulin, mU/L	11.90 ± 6.26
HbA1c, %	5.15 ± 0.23
HOMA index, mIU × mmol	2.30 ± 1.35
Renal function	
Creatinine, mg/dl	0.84 ± 0.14
GFR, ml/min per 1.73 m^2^	86.34 ± 12.35
Dietary habits	
DASH Score^a^	2.59 ± 1.37
Fruits and vegetables	553 (29.1)
Fish	330 (17.4)
Whole grains	779 (41)
Sodium	932 (49.1)
Sugar-sweetened beverage	1,134 (59.7)
Nuts	568 (29.9)
Meat	586 (30.8)
AFHC Score	10.69 ± 3.97

Values are given as mean ± SD and *n* (%); BMI, body-mass-index; BP, blood pressure; HDL-C, high-density lipoprotein cholesterol; LDL-C, low-density lipoprotein cholesterol; BP, blood pressure; GFR, glomerular filtration rate.

^a^ DASH Score components are based on the following recommendations for dietary intake: fruits and vegetables: ≥4 cups (portions) per day; fish: ≥two-times fish per week; whole-grain products: ≥3 servings per day; sodium: <1.5 g per day; sugar-sweetened beverages: <450 kcal/week; nuts and legumes: ≥4 portions per week; meat: no or max. 2 portions per week.

**Figure 1:**
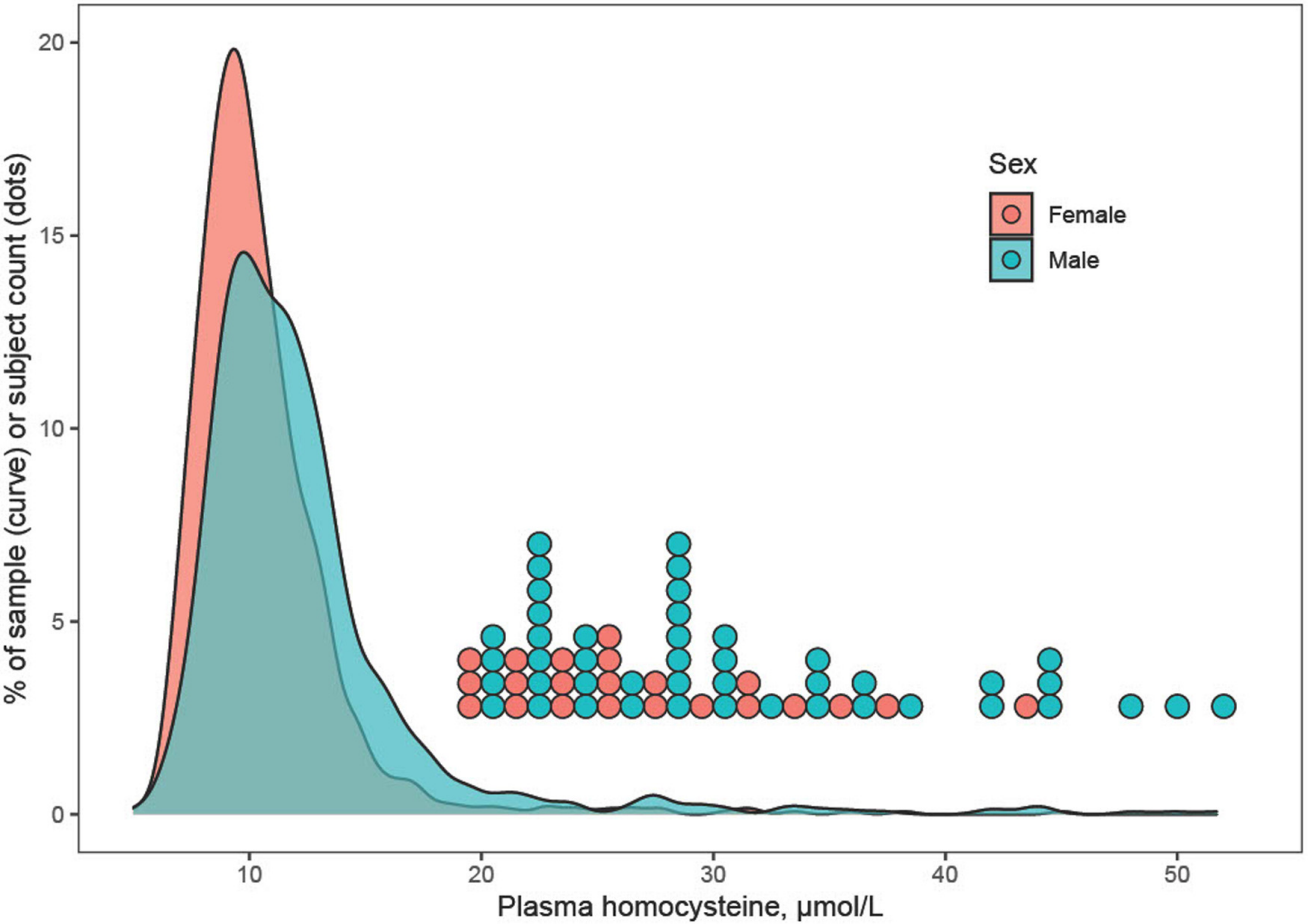
Distribution of plasma homocysteine by sex. The distribution of homocysteine (Hcy) showed extreme right-skew, which may indicate a mixture distribution including carriers of methylenetetrahydrofolate reductase or other mutations that associate with elevated Hcy. Males tended to feature higher Hcy than females, and this was true for subjects with Hcy ≤ 97th percentile as well as for subjects with very high Hcy > 97th percentile (corresponding to >20 µmol/L). For the latter, individual data points are shown as a dotplot histogram.

**Figure 2:**
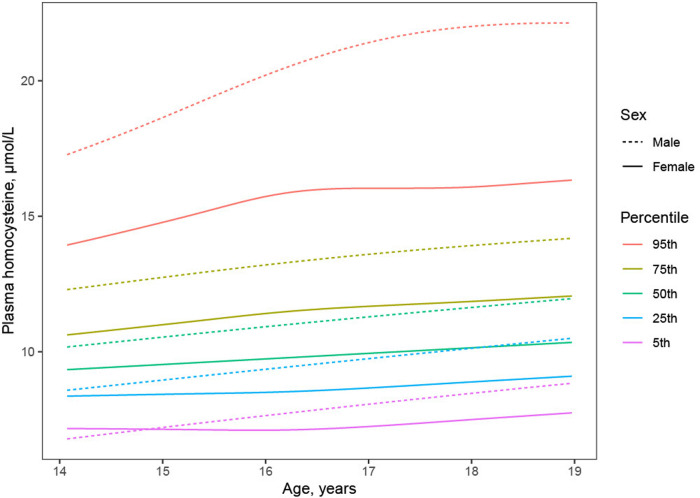
Trajectory of plasma homocysteine during adolescence by sex. Although males (dashed lines) compared to females (solid lines) showed higher average Hcy (black lines) throughout adolescence, investigation of Hcy percentiles conditional on age (colored lines) revealed higher male Hcy at the high end of the distribution at all ages, faster increase of Hcy in males than in females, and plateauing of Hcy in females but not in males around age 16.

Plasma Hcy showed a distinct distribution characterized by extreme right-skew (Figure 1). Average Hcy was 11.3 ± 4.48 µmol/L, and males featured higher Hcy than females overall and among subjects with lower Hcy ≤ 97th percentile (corresponding to 20 µmol/L) as well as among subjects with Hcy > 97th percentile. The sex difference was accentuated at the high range of Hcy, and increased with age (Figure 2). Although males showed a larger increase during the course of adolescence, in both males and females the increase slowed around age 16 (Figure 2). Reference percentiles for Hcy are shown in Table 2.

**Table 2:** Percentiles of total plasma homocysteine levels in µmol/L according to sex and age in years.

Sex	Age	5th	25th	50th	75th	95th
Female	14–15	7.2	8.4	9.4	10.8	14.3
Female	15–16	7.1	8.5	9.6	11.2	15.3
Female	16–17	7.2	8.6	9.9	11.6	16.0
Female	17–18	7.4	8.8	10.1	11.8	16.0
Female	18–19	7.6	9.0	10.3	12.0	16.2
Male	14–15	7.0	8.8	10.4	12.5	17.9
Male	15–16	7.4	9.2	10.7	13.0	19.5
Male	16–17	7.9	9.6	11.1	13.4	20.9
Male	17–18	8.3	10.0	11.5	13.8	21.8
Male	18–19	8.7	10.3	11.8	14.1	22.1

Factors associated with Hcy were numerous among the candidate clinical and laboratory parameters examined (Table 3). Direct associations with Hcy emerged for age, creatinine, BMI, systolic and diastolic BP, whereas inverse associations were detected for GFR and HDL-C as well as laboratory measures of glucose metabolism (HbA1c, insulin, and HOMA-IR). Better diet quality as assessed by the DASH and AFHC scores was likewise inversely associated with Hcy, and Hcy was lower with higher fruit intake but higher with higher meat and sweet beverage intake (Table 3).

**Table 3:** Univariate spearman correlation analysis of homocysteine with clinical/laboratory characteristics.

Characteristics	total	*p*-value	males	*p*-value	females	*p*-value
Demographics and anthropometrics						
Sex	−0.25	<0.0001*				
Age, years	0.13	<0.0001*	0.15	<0.0001*	0.11	<0.001*
BMI, kg/m^2^	0.05	0.042*	0.02	0.633	0.06	0.054
Lifestyle factors						
Smoking, %	0.02	0.329	−0.03	0.370	0.08	0.010*
Physical activity	0.04	0.064	−0.07	0.031*	0.01	0.635
Hemodynamics						
BP systolic, mmHg	0.17	<0.0001*	0.08	0.020*	0.07	0.033*
BP diastolic, mmHg	0.05	0.040*	0.07	0.053	0.06	0.047*
Lipid parameters						
Total cholesterol, mg/dl	−0.04	0.051	0.03	0.445	0.05	0.076
HDL-C, mg/dl	−0.05	0.027*	0.00	0.903	0.07	0.017*
LDL-C, mg/dl	−0.02	0.498	0.03	0.403	0.04	0.202
Glucose metabolism						
Glucose, mg/dl	−0.04	0.124	−0.13	<0.001*	−0.07	0.032*
Insulin, mU/L	−0.06	0.008*	−0.04	0.232	−0.04	0.168
HbA1c, %	−0.05	0.017*	−0.07	0.055	−0.07	0.017*
HOMA index, mIU × mmol	−0.06	0.006*	−0.07	0.064	−0.05	0.082
Renal function						
Creatinine, mg/dl	0.34	<0.0001*	0.28	<0.0001*	0.21	<0.0001*
GFR, ml/min per 1.73 m^2^	−0.28	<0.0001*	−0.24	<0.0001*	−0.19	<0.0001*
Dietary habits						
DASH Score	−0.06	0.021*	−0.00	0.896	−0.03	0.287
Fruits and vegetables	−0.08	<0.001*	−0.04	0.311	−0.06	0.067
Fish	0.02	0.356	0.03	0.395	−0.02	0.524
Whole grains	0.02	0.347	0.00	0.983	0.03	0.298
Sodium	−0.02	0.328	−0.00	0.979	−0.04	0.244
Sugar beverage	−0.05	0.020*	−0.00	0.973	−0.00	0.941
Nuts	0.02	0.418	0.02	0.494	−0.02	0.564
Meat	−0.10	<0.0001*	−0.02	0.531	−0.04	0.221
AFHC Score	−0.09	<0.0001*	−0.04	0.325	−0.04	0.200

* (*p* < 0.05), Abbreviations and detailed description of the DASH score as Table 1.

Joint independent associated factors with Hcy were determined using L1-regularized linear regression after a normalizing transformation of the Hcy distribution. This analysis indicated that the strongest multivariable correlates of Hcy were sex and creatinine. Further variables selected by the LASSO were AFHC score, diastolic and systolic BP, HbA1c, HOMA index, age and sweet beverages, but these associations were not statistically significant (Table 4).

**Table 4:** Multivariable associations between homocysteine and clinical/laboratory characteristics.

Characteristics	Beta coefficient	*p*-value	LASSO coefficient
Creatinine, mg/dl	0.24 (0.19, 0.30)	<0.001	0.32
Sex	−0.21 (−0.33, −0.09)	<0.001	−0.20
AFHC Score	−0.05 (−0.09, 0.00)	0.056	−0.04
BP diastolic, mmHg	0.05 (−0.00, 0.10)	0.062	0.04
HbA1c, %	−0.05 (−0.09, 0.00)	0.051	−0.04
Age, years	0.03 (−0.02, 0.07)	0.292	0.03
HOMA Index, mIU × mmol	−0.04 (−0.09, 0.01)	0.107	−0.02
BP systolic, mmHg	0.03 (−0.03, 0.08)	0.403	0.02
Sweet beverages, %	−0.02 (−0.06, 0.03)	0.520	−0.01

Abbreviations are as in Table 1. Effects are for female vs. male sex for sex, and for a 1-standard deviation higher level for other variables. All depicted variables were selected by LASSO with effect sizes as shown in the “LASSO coefficient” column, and selection was driven by predictive performance. Selected variables were additionally included in a conventional linear regression model to obtain confidence intervals and significance levels, as shown in the “Beta coefficient” and “*p*-value” columns.

The following variables were not selected by LASSO: Dietary intake of fruits, of meats, insulin levels, body mass index, glomerular filtration rate, and HDL cholesterol.

Effects are shown for a 1-SD increase vs. the reference category.

67 participants with Hcy above the 97th percentile were invited for further in-house check-up, 38 participated, with consent to genetic testing available for 13. None of these 13 were negative for the MTHFR polymorphism (c.665C > T), two were heterozygous, and 11 were homozygous.

## 4. Discussion

This large-scale observational study describes plasma Hcy distributions and trajectories during adolescence and defines its important univariable and multivariable associated factors. Key findings include (i) a distinct Hcy distribution characterized by extreme right-skew, (ii) sex-differential age trajectories of Hcy, and (iii) a paramount dependence of Hcy on sex and creatinine with lesser importance of a host of other anthropometric, lifestyle, dietary, and laboratory parameters.

The distribution of plasma Hcy was similar, and average Hcy moderately higher than previously reported in adolescent cohorts (Figure 1) (11, 23–25) and Hcy increased with age. Although increases of Hcy during adolescence, as during childhood and during adulthood have been reported, the current study describes a greater increase in males and a plateauing of Hcy in particular in subjects with high Hcy around age 16 (Figure 2), refining prior findings of higher Hcy levels in boys than in girls (4, 26, 27).

Possible mechanisms underlying sex differences in Hcy include the enzyme cysthathionine β synthase (CBS), a key enzyme in the transsulfuration pathway that facilitates conversion of Hcy to cysteine. Testosterone-dependent CBS downregulation may be one cause of higher Hcy in males (28, 29). Another cause may lie in creatine metabolism. Guanidinoacetate methyltransferase is an enzyme that metabolizes guanidinoacetate to creatine, resulting in higher Hcy production. Creatinine is further processed by nonenzymatic degration of creatine. Males, due to higher muscle mass, feature higher creatine turnover rates and consequently higher production of Hcy (11, 30). In line, we found a significant association of Hcy with creatinine (Table 3), which was among the strongest univariable and multivariable predictors of Hcy (Table 4).

Impaired renal clearance of Hcy further links creatinine and GFR to HHcy. Increases in Hcy have been demonstrated in children receiving renal replacement therapy and children with impaired renal function after kidney transplantation (31). A recent study investigated the relationship between homocysteine and serum uric acid in a 12- to 19-year-old cohort from the NHANES study in the U.S (32). Likewise, they found a strong correlation between low GFR and high homocysteine levels, however didn't investigate independent effects of GFR on homocysteine in this adolescent cohort. Yet, the Bogalusa Heart Study in community-based 24- to 42-year-old young adults found an independent adverse relationship of GFR with homocysteine (33). It can be cautiously assumed that independent effects of GFR on homocysteine might become apparent with decreasing GFR and with increasing age.

Further univariable associated factors with Hcy (Table 3) included systolic and diastolic BP, whereas inverse associations were detected for laboratory measures of glucose metabolism (HbA1c, insulin, and HOMA-IR) and dietary habits.

The associations of systolic and diastolic blood pressures with Hcy found here are consistent with prior studies in adolescent and adult cohorts (12, 13, 23, 34). Although one prior study reported a U-shaped relation between BP and Hcy, indicating that high as well as low Hcy may affect BP (11), we could not corroborate this finding (not shown). It has been hypothesized that high Hcy causes vascular dysfunction by inducing oxidative stress, followed by endothelial dysfunction and vascular remodeling resulting in arterial hypertension (35). Previous investigations have described a causal relationship of HHcy and subclinical early vessel pathologies in children with comorbidities such as obesity or hypertension (7, 36).

To the best of our knowledge, this study is the first to describe an inverse association between Hcy and HbA1c. So far, only one smaller cohort of 12- to 16-year old adolescents investigated HbA1c in relation to Hcy and found no significant relationship (12). We detected moderate univariate inverse correlations of Hcy with insulin and HOMA index. According to the literature, a relationship between Hcy and hyperinsulinemia is controversially debated (37, 38). Our findings stand in line with previous investigations, which demonstrated that acute hyperinsulinemia leads to a decrease of plasma Hcy levels in non-diabetic patients (39). In contrast, patients with insulin resistance and type 2 diabetes show higher homocysteine levels. It has been suggested that insulin modulates CBS activity, and modulation becomes disturbed in insulin resistance (40). This hypothesis would be consistent with the results of a study in Austrian obese children and adolescents that identified fat mass-associated hyperinsulinism as a contributor to HHcy (41).

So far, few studies have investigated the association between dietary habits and Hcy in adolescents (42, 43). Better diet quality as assessed by the DASH and AFHC scores was likewise inversely associated with Hcy, and Hcy was lower with higher fruit intake but higher with higher meat and sweet beverage intake (Table 3). This is in line with prior reports of amelioration of Hcy levels after modification of diet towards a DASH-style diet (44). The inverse association between Hcy and meat intake that we found is consistent with prior findings in adults and adolescents of a direct association between high intake of animal protein and HHcy. Methionine is the only dietary precursor of Hcy and is mainly found in red meats, poultry, fish, dairy products, and eggs. Hence, high methionine consumption may directly result in high Hcy levels (43, 45). In our study, low fruit and vegetable intake was associated with Hcy. Likewise, a beneficial effect of fruit and vegetable intake on Hcy levels has been described in adult cohorts, possibly explained by a higher intake of micronutrients, in particular folate (46). Overall, associations of dietary components with Hcy were modest. Further detailed analyses of micronutrient intakes and their relation to Hcy in this study cohort are planned.

Strengths of this study include the large, representative, well-characterized study population that was suited to determine plasma Hcy levels and associated factors in healthy community-dwelling adolescents. Limitations include that vitamin status of participants was unknown, although Hcy metabolism is known to depend on vitamins B-6, B-12 and on folate levels. Moreover, complete information on the MTHFR polymorphism, which is a predisposing factor for HHcy, was only available in a very small subset of our sample and is therefore not further discussed (47).

When addressing the cardiovascular risk of homocysteine reduced, free-oxidized and protein-bound forms of Hcy, cysteine and cysteinylglycerine referred to as redox thiol status should be considered in future studies (48). Studies in adults have shown that increased oxidative stress measured by the concentration of oxidized and reduced thiol markers might predict early atherosclerosis (49, 50). Hence, associations of oxidized and reduced forms of Hcy with cardiovascular risk factors should be further taken into account in adolescent cohort studies on the vascular risk of homocysteine.

In summary, this study defines clinical and laboratory factors associated with plasma Hcy levels in adolescents with sex and creatinine showing the strongest independent effects on Hcy. Some previously described risk factors of Hcy in pediatric and adult populations, including high systolic blood pressure and insulin resistance, could not be confirmed as independent associated factors in this cohort of healthy adolescents. Taken together, these results may aid when interpreting future studies on the vascular risk of homocysteine.

## Data Availability

The raw data supporting the conclusions of this article will be made available by the authors, without undue reservation.
