# Supplementary material for: Plasma homocysteine levels and associated factors in community-dwelling adolescents: the EVA-TYROL study
Source: Front Cardiovasc Med. 2023 Jun 23;10:1140990. doi: 10.3389/fcvm.2023.1140990 (PMC10327549; doi:10.3389/fcvm.2023.1140990)
Supplement: Supplementary file 1 [file Datasheet1.pdf]

Supplementary material

Plasma homocysteine levels and associated factors in community-dwelling adolescents: The EVA-TYROL Study

Nina Gande[1], Christoph Hochmayr[1], Anna Staudt[1], Benoît Bernar[2], Katharina Stock[3], Sophia J. Kiechl[4], Ralf Geiger[3,5], Andrea Griesmacher[6], Sabine Scholl-Bürgi[2], Michael Knoflach[4], Raimund Pechlaner[4*], Ursula Kiechl-Kohlendorfer[1], and The (Early Vascular Ageing) EVA Study Group.

[1] Department of Pediatrics II (Neonatology), Medical University of Innsbruck, Innsbruck, Austria

[2] Department of Pediatrics I, Medical University of Innsbruck, Innsbruck, Austria

[3] Department of Pediatrics III (Cardiology), Medical University of Innsbruck, Innsbruck, Austria

[4] Department of Neurology, Medical University of Innsbruck, Innsbruck, Austria

[5] Department of Pediatrics, Bruneck Hospital, Bruneck, Italy

[6] Central Institute of Clinical Chemistry and Laboratory Medicine Medical University of Innsbruck, Innsbruck Austria

[*]Corresponding author at: Department of Neurology, Medical University of Innsbruck, Innrain 52, Innsbruck, Austria. Email address: raimund.pechlaner@i-med.ac.at (R. Pechlaner)

1 Early Vascular Ageing (EVA) Study Group:

Mandy Asare[4]

Manuela Bock-Bartl[4]

Maximilian Bohl[1]

Christina Schreiner[1]

Gregor Brössner[4]

Tatjana Heisinger[1]

Julia Klingenschmid[1]

Martina Kothmayer[1]

Julia Marxer[1]

Maximilian Pircher[1]

Carmen Reiter[1]

Bernhard Winder[1]
